# Evaluation of urinary L-FABP as an early marker for diabetic nephropathy in type 2 diabetic patients

**DOI:** 10.2478/jomb-2019-0037

**Published:** 2020-01-23

**Authors:** Thuy Ngan Duong Thi, Binh Nguyen Gia, Huong Lan Le Thi, Thu Nguyen Cuc Thi, Huong Phung Thanh

**Affiliations:** 1 Thai Nguyen Medical College, Thai Nguyen, Vietnam; 2 Vinmec Hospital, Hanoi, Vietnam; 3 A Hospital, Thai Nguyen, Vietnam; 4 Hanoi University of Pharmacy, Hanoi, Vietnam

**Keywords:** albuminuria, diabetic nephropathy, type 2 diabetes, urinary L-FABP, urinarni L-FABP, dijabetes tip 2, dijabetička nefropatija, albuminurija

## Abstract

**Background:**

Albuminuria is the standard biomarker for the diagnosis of diabetic nephropathy (DN). However, some patients with persistent microalbuminuria still progress to chronic kidney disease, raising the question of finding a better biomarker. This study aimed to evaluate the correlation of urinary liver-type fatty acid-binding protein (L-FABP) levels with renal function and to compare the role of urinary albumin-to-creatinine ratio (ACR) with urinary L-FABP in early detection of DN in type 2 diabetic patients.

**Methods:**

The cross-sectional study was done on 106 type 2 diabetic patients and 30 non-diabetic people. L-FABP was measured with the Latex enhanced immunoturbidimetric technique.

**Results:**

There was a strong and negative correlation between the urine L-FABP levels and eGFR (r = -0.606, p<0.001). The urinary L-FABP levels were significantly higher (p<0.001) in the normoalbuminuria diabetic group than the non-diabetic control group. The ROC-curve analyses in the diabetic patients and the normoalbuminuria diabetic patients showed that the AUCL-FABP was remarkably higher (p<0.001) than the AUCACR. An optimal cutoff value of 5 mg L-FABP/g Cr (with the sensitivity of 98.1% and specificity of 90%) and of 4.3 mg L-FABP/g Cr (with the sensitivity of 100% and specificity of 86.67%) was set to detect DN in the diabetic patients and the normoalbuminuria diabetic patients, respectively.

**Conclusions:**

The change in urinary L-FABP levels happened earlier than in urinary albumin during renal function impairment. Urinary L-FABP can be used as a better indicator than ACR for early detection of DN in type 2 diabetes.

## Abstract (sr)

**Uvod:**

Albuminurija je standardni biomarker za dijagnozu dijabetičke nefropatije (DN). Međutim, kod nekih pacijenata sa perzistentnom mikroalbuminurijom dolazi do hronične bolesti bubrega, pa se postavlja pitanje pronalaženja boljeg biomarkera. Ova studija je imala za cilj da proceni korelaciju nivoa jetrenog tipa vezujućeg proteina za masne kiseline (L-FABP) sa funkcijom bubrega i da uporedi ulogu urinarnog odnosa albumin-kreatinin (ACR) sa urinarnim LFABP u ranom otkrivanju DN kod pacijenata sa dijabetesom tipa 2.

**Metode:**

Sprovedena je studija preseka na 106 bolesnika sa dijabetesom tipa 2 i 30 osoba koje nisu dijabetičari. L-FABP je meren Lateks pojačanom imunoturbidimetrijskom tehnikom.

**Rezultati:**

Postoji jaka i negativna korelacija između nivoa L-FABP urina i eGFR (r = -0,606, p < 0,001). Nivoi LFABP u urinu su bili značajno viši (p < 0,001) u grupi dijabetičara normoalbuminurije nego u kontrolnoj grupi bez dijabetesa. Analiza ROC krive kod dijabetičara i kod pacijenata sa dijabetesom normoalbuminurije pokazala je da je AUCL-FABP značajno veći (p < 0,001) od AUCACR. Postavljena je optimalna granična vrednost od 5 mg L-FABP/g Cr (sa osetljivošću 98,1% i specifičnosti 90%) i 4,3 mg LFABP/g Cr (sa osetljivošću od 100% i specifičnosti od 86,67%) da bi se detektovala DN kod dijabetičara i normalno albuminurijskih dijabetičara, posebno.

**Zaključak:**

Promena nivoa L-FABP u urinu se desila ranije nego u urinarnom albuminu tokom oštećenja bubrežne funkcije. Urinarni L-FABP se može koristiti kao bolji indikator od ACR-a za rano otkrivanje DN kod dijabetesa tipa 2.

## Introduction

The prevalence of diabetes mellitus, a worldwide burden, is expected to affect more than 350 million people by 2035 [1]. About one-third of diabetic patients have microalbuminuria after 15 years of disease duration, and nearly half of them develop real kidney disease, a serious complication with negative impacts on health, quality of life and even life expectancy [2] [3]. Early detection and intervention are essential in the prevention and treatment of diabetic nephropathy (DN). So far, albuminuria or the urinary albumin-to-creatinine ratio (ACR) has been the standard marker for early detecting DN as recommended by various guidelines and reports. However, there have been controversies over its diagnostic and prognostic significance since, in several studies, macroalbuminuria accompanied rather than preceded progression to advanced chronic kidney disease (CKD). Furthermore, patients with persistent microalbuminuria still progress to CKD stages 3-5 [4]. Therefore, it is necessary to find novel biomarkers with better specificity and sensitivity to effectively detect and intervene in DN at the onset for better prevention of CKD progression.

Liver-type fatty acid-binding protein (L-FABP) is an intracellular fatty acid carrier protein which expresses mainly in the liver and kidney. L-FABP was supported to be associated with renal tubulointerstitial damage due to excessive reabsorption of free fatty acids [5]. Determination of plasma and especially urinary L-FABP have been reported in recent studies as potential biomarkers for early diagnosis of acute kidney injury (AKI) caused by various factors such as after cardiopulmonary bypass surgery [5], after cardiac surgery [6] or in critically ill patients [7].

This cross-sectional descriptive study aims to evaluate the correlation of urinary L-FABP levels with renal function and to compare the role of ACR with urinary L-FABP in early detection of DN in type 2 diabetic patients.

## Materials and Methods

### Patients

Type 2 diabetic patients between 18 and 80 years old admitted into the A Hospital (Thai Nguyen province, Vietnam) from August 2018 to November 2018 were enrolled in the study. The exclusion criteria were cardiovascular diseases in the last three months, uncontrolled hypertension, severe infections in the last three months, current acute infections, pregnant women, patients with liver diseases, patients with end-stage renal disease, unwillingness to participate in the study. A total of 106 type 2 diabetic patients and a control group of 30 non-diabetic people (recruited from the Outpatient Department of the same hospital) were enrolled in the study. All of them provided their informed written consent. The study complied to the Declaration of Helsinki and was approved by the Ethics Committee of the A Hospital with approval number BVA-2018-07-2.

Type 2 diabetic patients were divided into three subgroups based on their renal status. Group 1 (n=41) included the normoalbuminuria subjects with ACR of <30 μg/mg creatinine, group 2 (n=47) included the microalbuminuria subjects with ACR from 30 to 300 μg/mg. Group 3 (n=18) comprised of the macroalbuminuria subjects with ACR >300 μg/mg.

### Data collection

Fasting serum samples were used for the estimation of biochemical parameters, including creatinine and glucose. The glomerular filtration rate (GFR) was estimated using the Modification of Diet Renal Disease equation.

Fresh urine samples were collected in the early morning for measurement of urinary creatinine and albumin, using CLINITEX Novus urine analyzer. 3mL of the fresh urine sample was centrifuged at 2000 rpm for 10 minutes. The supernatant was stored at -80 °C until tested. L-FABP levels were measured with the Latex enhanced immunoturbidimetric technique, using NORUDIA™ L-FABP kit (Sekisui Medical Co., Ltd, Japan) on the AU480 automated analyzer (Beckman Coulter). Urinary L-FABP levels were expressed as values adjusted for the urinary creatinine levels (μg/g Cr).

### Statistical analysis

Continuous variables were expressed as mean±SD and compared using one-way ANOVA with a normal distribution. Median (interquartile range) and one-way Kruskal-Wallis were alternatively used for nonparametric distribution. The correlation of continuous variables was tested with Pearson's or Spearman's formula depending on the distribution.

The correlation between L-FABP and eGFR was shown by the scatter plot and determined by regression analysis. The distribution of L-FABP between stages was also emulated by box plot diagram. The clinical benefits of using L-FABP and ACR were assessed with Receiver operating characteristic (ROC) curve analyses. P-value <0.05 was considered as statistical significance. R software Version 3.5.1 was applied for all statistical analyses.

## Results

### Characteristics of study subjects

The general characteristics and laboratory data of the study subjects were demonstrated in Table 1. There was no difference in gender among the subjects (p=0.062), but the diabetic patients had higher ages than the control (p<0.001). There was a gradual increase of both systolic and diastolic blood pressure, HbA1c, glucose, triglyceride, urinary L-FABP, and ACR in parallel with a gradual decrease of eGFR among the study groups from the nondiabetic subjects to the type 2 diabetic patients of various stages of albuminuria (p<0.001). There was a significant difference in total cholesterol among the study groups (p<0.001).

**Table 1:** Characteristics of study subjects Categorical data are presented as numbers, and continuous data are presented as median (interquartile range)

	Control (n=30)	Type 2 diabetic patient groups			p
		Normoalbuminuria (n=41)	Microalbuminuria (n=47)	Macroalbuminuria (n=18)	
Gender					0.062
Males	10	22	24	4	
Females	20	19	23	14	
Age (years)	35.5 (28)	59.00 (10)	60.00 (10)	59.00 (15.00)	0.000
Blood pressure (mm Hg) Systolic Diastolic	110 (10) 70 (10)	120 (20) 70 (10)	130 (30) 80 (20)	140 (20) 90 (13)	0.000 0.000
HbA1c (%)	4.75 (1.35)	7.00 (1.00)	7.50 (2.20)	8.80 (1.73)	0.000
Glucose (mmol/L)	4.80 (1.37)	7.80 (4.55)	9.20 (4.70)	12.55 (3.78)	0.000
Total cholesterol (mmol/L)	4.15 (1.08)	5.00 (1.40)	5.30 (1.60)	5.20 (1.73)	0.000
Triglyceride (mmol/L)	1.56 (0.57)	2.71 (1.56)	2.87 (1.05)	3.60 (1.42)	0.000
eGFR (mL/min/1.73 m^2^)	102.5 (9)	95.00 (6.00)	72.00 (35.00)	52.50 (14.00)	0.000
L-FABP (μg/g Cr)	1.99 (1.91)	6.30 (2.30)	6.77 (4.79)	18.07 (38.69)	0.000
ACR (mg/g Cr)	27.61 (4.73)	27.47 (7.46)	164.67 (95.8)	433.10 (184.75)	0.000

### Association of urinary L-FABP and renal function

**Figure 1:**
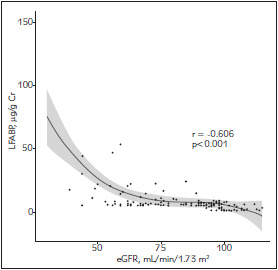
Association between the urinary L-FABP levels and eGFRs

A Spearman's correlation between the urinary L-FABP levels and eGFRs were analyzed (Figure 1). There was a strong and negative correlation between the urine L-FABP levels and eGFR (r=-0.606, p<0.001).

**Figure 2:**
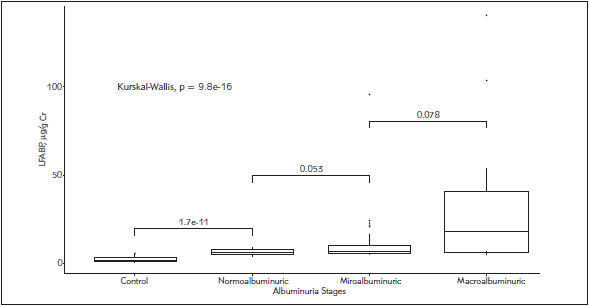
Comparison of urinary L-FABP levels among the study groups

The urinary L-FABP levels were significantly higher (p<0.001) in the normoalbuminuria group than the non-diabetic control and the macroalbuminuria group than the microalbuminuria group. There was no significant difference in the urinary L-FABP levels between the microalbuminuria and the macroalbuminuria group (Figure 2).

### Comparison of the role of ACR with urinary L-FABP in early detection of DN in type 2 diabetic patients

**Figure 3:**
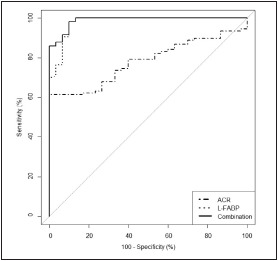
ROC curve analysis for L-FABP and ACR in type 2 diabetic patients

A ROC-curve analysis for urinary L-FABP and ACR in type 2 diabetic patients (Figure 3 and Table 2) showed that the AUC_L-FABP_ was notably higher (D_AUCs_ = 0.199, p<0.001) than the AUC_ACR_. An optimal cutoff value of 5 μg L-FABP/g Cr was set with the sensitivity of 98.1% and specificity of 90%. The combination of L-FABP and ACR did not significantly increase the AUC compared to a single use of L-FABP (p=0.171).

**Table 2:** The area under the ROC curves of urinary L-FABP and ACR in type 2 diabetic patients * in comparison with L-FABP

	AUC-ROC (95% CI)	P
ACR (mg/g Cr)	0.78 (0.70–0.86)	0.000*
L-FABP (μg/g Cr)	0.98 (0.95–1.00)	–
Combination	0.99 (0.97–1.00)	0.171*

**Figure 4:**
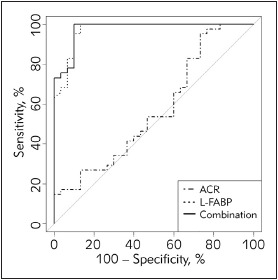
ROC curve analysis for L-FABP and ACR in normoalbuminuria diabetic patients

A ROC-curve analysis for urinary L-FABP and ACR in normoalbuminuria diabetic patients (Figure 4 and Table 3) showed that the AUC_L-FABP_ was remarkably higher (D_AUCs_ = 0.401, p<0.001) than the AUC_ACR_. An optimal cutoff value of 4.3 mg L-FABP/g Cr was set with the sensitivity of 100% and specificity of 86.67%. The combination of L-FABP and ACR did not significantly increase the AUC compared to a single use of L-FABP (p=0.318).

**Table 3:** The area under the ROC curves of urinary L-FABP and ACR in normoalbuminuria diabetic patients * in comparison with L-FABP

	AUC-ROC (95% CI)	p
ACR (mg/g Cr)	0.57 (0.43–0.71)	0.000*
L-FABP (μg/g Cr)	0.97 (0.94–1.00)	–
Combination	0.98 (0.95–1.00)	0.318*

### Discussions

Although urinary L-FABP was confirmed as a newly established tubular biomarker by the Ministry of Health, Labour and Welfare in Japan in 2010, there has not been much consistent clinical evidence of the association between L-FABP and renal function, especially in diabetic patients as reviewed by Kamijo-Ikemori et al. [8]. This is the first study of the clinical significance of urinary L-FABP in Vietnamese type 2 diabetic patients. Our results in Figure 1 showed that urinary L-FABP levels increased in parallel with the eGFR declines (r =-0.606, p<0.001). The strong correlation between urinary L-FABP levels and the eGFR was also demonstrated on type 2 diabetic patients in a study by Suzuki et al. [9] and by Kare et al. [10].

The association between the urinary L-FABP levels and the renal function was supported by the differences in the urinary L-FABP levels among the various albuminuria stages (Figure 2). There was a significant difference (p<0.001) of the urinary L-FABP levels among the study groups. Similar to those reported by Suzuki et al. [9], the urinary L-FABP levels were significantly higher (p<0.001) in the macroalbuminuria group than the microalbuminuria group. It has been well known that the tubular system played an important role in the pathophysiology of DN. The diabetic milieu and the prolonged interactions of albuminuria, advanced glycation end products (AGEs) and other factors in the glomerular filtrate with the tubular system induce renal oxidative stress and cortical interstitial inflammation, resulting in hypoxia and tubulointerstitial fibrosis which lead to the DN progression [11] [12] [13]. That is the essential reason why the high urinary L-FABP levels were found in those with impaired renal function in our results.

Notably, the urinary L-FABP levels of diabetic patients with normoalbuminuria were significantly higher (p<0.001) than those of the normal control, suggesting that urinary L-FABP is an earlier indicator than urinary albumin in the detection of renal disorders in diabetic patients. The early change of urinary L-FABP levels in type 1 diabetic patients with normoalbuminuria was also reported by Panduru et al. [14].

So far, microalbuminuria has been the standard marker for detection of DN. However, impaired renal function has been found in both type 1 and type 2 diabetic patients with normoalbuminuria [15] [16] [17], raising the question of the clinical significance of such well-known biomarker. Therefore, it is critical to find an alternative or adjuvant biomarker in addition to ACR for early detection of DN. The ROC analysis among the type 2 diabetic patients showed that the AUC_L-FABP_ was significantly higher than the AUC_ACR_. A higher value of AUC_L-FABP_ than AUC_ACR_ in predicting DN progression in type 2 diabetic patients was reported for the first time by Kamijo-Ikemori et al. [18]. The sensitivity and specificity of urinary L-FABP for diagnosis of DN in type 2 diabetes in our study (98.1% and 90%, respectively) were higher than those for diagnosis of AKI in a meta-analysis (74.5% and 77.6%, respectively). Such results of the ROC analysis suggested an advantage of using urinary L-FABP for diagnosing and monitoring DN in type 2 diabetic patients over its use in AKI. An optimal cutoff value of 5 μg L-FABP/g Cr was set with the sensitivity of 98.1% and specificity of 90%. We have not found any publication on the cutoff value of urinary L-FABP in the diagnosis of DN in type 2 diabetes yet. Much higher cutoff values of urinary L-FABP were set to monitor CKD (17.4 μg/g Cr) [19] or to predict contrast-induced AKI (24.5 μg/g Cr) [20]. In this study, the combination of L-FABP and ACR did not significantly increase the AUC compared to a single use of L-FABP. In a study on type 1 diabetic patients, the combination of L-FABP and albumin excretion rate did not improve the ROC-AUC compared with a single use of each parameter [14].

The advantage of urinary L-FABP over ACR is its earlier change, so we did a ROC analysis in the normoalbuminuria diabetic patients to evaluate the capability of early detection of DN. The results in Figure 4 showed the superiority of urinary L-FABP to ACR to detect renal dysfunction in the absence of albuminuria (D_AUCs_=0.401, p<0.001). The sensitivity and specificity of urinary L-FABP were 100% and 86.67%, respectively. An optimal cutoff value of 4.3 μg L-FABP/g Cr was set, that is a little bit lower than that in the total diabetic patients. These results support using urinary L-FABP as an effective indicator for early detection of DN, thereby could delay or halt the progression of renal impairment.

One of the most important limitations of this study was the non-matching in the age of the diabetic groups and the control group. However, as revealed by Kashiwagi et al. [21], no significant correlation was found between urinary L-FABP and age. That means the difference in age among the study subjects might not have any fundamental impacts on the analysis of L-FABP role.

Another limitation is the cross-sectional design of this study. However, the results of this study would be an essential base of a time and money-consuming comprehensive prospective cohort with long durations of follow-up for a better evaluation of L-FABP in predicting DN.

In conclusion, urinary L-FABP level strongly and negatively correlated to eGFR and increased with albuminuria severity. The change in urinary L-FABP levels happened earlier than in urinary albumin in impairment of renal function. Urinary L-FABP level can be used as a better indicator than ACR for early detection of DN in type 2 diabetes.

### Author Contributions

D.T.T.G collected and analyzed the data, N.G.B co-supervised the research and edited the manuscript, L.T.H.L discussed, contributed to the research and edited the manuscript. C.T.T.N did the statistical analysis and co-wrote the manuscript. P.T.H designed, co-supervised the research, and co-wrote the manuscript.

## Conflict of interest statement

The authors state that they have no conflicts of interest regarding the publication of this article.

## List of abbreviations

ACR, albumin-to-creatinine ratio; AGEs,advanced glycation end products; AKI, acute kidney injury; CKD, chronic kidney disease; DN, diabetic nephropathy; GFR, glomerular filtration rate; L-FABP, Liver-type fatty acid-bindingprotein; ROC, Receiver operating characteristic curve.
